# Food allergy and anaphylaxis – 2063. Identification of foods causing hypersensitivity/ allergy among school children in two sub-urban schools in Colombo District, Sri Lanka

**DOI:** 10.1186/1939-4551-6-S1-P146

**Published:** 2013-04-23

**Authors:** Y Sarangee G Wimalasiri, RM Udayangani Ratnayake, TDN Karunaratne, KKDS Ranaweera

**Affiliations:** 1 Ayurveda, Institute of Indigenous Medicine, University of Colombo, Colombo, Sri Lanka; 2 Department of Biochemistry, Faculty of Medicine & Allied Sciences, Rajarata University of Sri Lanka, Saliyapura, Sri Lanka; 3 Bandaranayake Memorial Ayurveda Research Institute, Nawinna, Colombo, Sri Lanka

## Background

In general, different foods and food ingredients are believed to be causing hypersensitivity/allergy among the Sri Lankan population. Global epidemiological observations have revealed lists and levels of hypersensitive foods but proper researches have been carried out in Sri Lanka for identifying food allergens. This study was carried out to obtain statistical data for different food categories causing hypersensitivity in school children in Colombo Districts, Sri Lanka.

## Methods

Descriptive cross sectional study was conducted for adolescences aged between 16 to 19 years selected from two sub-urban schools; Maharagama Educational division, Colombo District during July 2012. Study tool was self administered semi structured questionnaire. Statistical data analysis was performed by using statistical package SPSS 15.

## Results

A total number of 452 school children were recruited for the study and 449 had responded thus, response rate was 99.34%. From the respondents 137 (30.3%) had answered ‘yes’ for hypersensitivity reactions on foods, drugs, pollen, dust or any other allergen/agent. Out of the total hypersensitivity declared individuals, 90 (19.9%) respondents had food specific hypersensitivity.

Foods were grouped into seven categories such as Fruits, Vegetables, Fish and sea foods, Meat and eggs, Milk, Spices and ‘Other foods’.

From the hypertensive declared individuals most hypertensive food group was Fruits (58; 42.34%). The second frequent problematic food group was Fish and sea foods (52; 37.98%). Vegetables (40; 29.19%), ‘Other food’ category (29; 21.16%), Meat & eggs (24; 17.5%), Milk (6; 4.37%) and Spices (2; 1.46%) were found to be hypersensitive to the study population in specified percentages.

## Conclusions

Pineapple (49; 35.7%) was the most hypersensitivity fruit among adolescences studied. Generally in Sri Lanka people tend to believe that pineapple causes adverse reactions in the body and the gathered data supports the belief. Rambutan (*Nephelium lappaceum*) (27; 19.7%) was the next prevalent hypersensitive fruit.

Among the vegetables mostly tomato (25; 18.24%) and bread fruit (*Artocarpus altilis*) (14; 10.21%) and from fish & sea food category, prawns (27; 19.7%), cuttle fish 24 (17.5%), tuna (24; 8.75%) and canned fish (18; 13.13%) were found to be hypertensive to the study population.

